# Safety and feasibility of a 7F thin-walled sheath *via* distal transradial artery access for complex coronary intervention

**DOI:** 10.3389/fcvm.2022.959197

**Published:** 2022-10-12

**Authors:** Bin Zong, Yi Liu, Bing Han, Chun-Guang Feng

**Affiliations:** Department of Cardiology, Xuzhou Central Hospital, Xuzhou, China

**Keywords:** distal radial artery, 7F thin-walled sheath, percutaneous coronary intervention (PCI), ultrasound, cardiology

## Abstract

**Background:**

Compared with traditional trans-radial artery access (TRA), there are limited data that can confirm the efficacy and safety of a 7F thin-walled sheath placed *via* distal TRA (dTRA) for percutaneous coronary intervention (PCI).

**Objective:**

This study aims to analyze the safety and efficacy of the placement of a 7F thin-walled sheath *via* dTRA for PCI.

**Methods:**

This was a single-center retrospective observational study in which 102 patients who received complex PCIs with a 7F thin-walled sheath placed *via* dTRA in the catheter room of our hospital from May 2020 to October 2021 were included. The basic information, puncture success rate, radial artery occlusion (RAO) rate, radial artery lumen diameter and area, surgical data, pain score, and complication rate were observed and recorded.

**Results:**

The puncture success rate was 90.2% in the 102 patients, and the success rate of the operation was 97.8% among 92 patients with a successful puncture. The PCIs for patients included emergency PCIs and all types of complex PCIs. Color Doppler ultrasound performed at 1 and 30 d after the procedure showed that the RAO rate was 2.2%, the distal RAO rate was 3.3%, the postoperative average pain score was 2.2 points, and there were five patients (5.4%) with local hematoma, all of which were grade 1–2. Radial artery spasm and nervous injury occurred in two patients (2.2%), and arteriovenous fistula occurred in one patient (1.1%). Radial artery perforation, radial artery dissection, pseudoaneurysm, and sheath kinking did not occur.

**Conclusion:**

The placement of a 7F thin-walled sheath *via* dTRA for PCI showed a high puncture and procedural success rate, a low postoperative RAO rate, and a low incidence of local hematoma and other complications. The placement of a 7F Glidesheath Slender^®^ *via* dTRA for PCI is safe and feasible.

## Key point

- PCI with the placement of a 7F thin-walled sheath *via* dTRA is characterized by a high success rate in puncture and procedure.
- A low postoperative RAO rate and a low incidence of local hematoma and other complications.
- PCI with the placement of a 7F thin-walled sheath *via* dTRA is safe and feasible.

## Introduction

With the continuous development of coronary artery treatment technology, transradial artery access (TRA) has been proven to be superior to transfemoral artery access. Compared with the latter, TRA can significantly reduce vascular complications such as bleeding, improve patient comfort, and even reduce mortality in patients with acute coronary syndrome (1). Therefore, more than 90% of coronary angiogram (CAG) and percutaneous coronary intervention (PCI) procedures are currently performed *via* TRA in China. However, radial artery occlusion (RAO) caused by TRA has gradually been recognized clinically, and the reported incidence of occlusion is 0–33%, limiting the repeated use of the radial artery for operating procedures (2, 3). The relative inconvenience of the TRA operation on the left side is also often mentioned (4). In 2017, Kiemeneij (4) first reported a clinical study on coronary intervention *via* distal TRA (dTRA) on the left side of the anatomical snuffbox, and, consequently, dTRA has gradually attracted attention. Some subsequent clinical observations also revealed that dTRA was characterized by a better patient experience, relatively short postoperative compression time, and a significantly reduced incidence of RAO;(4–7) thus, an increasing number of interventional cardiologists and treatment centers began to choose dTRA in coronary artery intervention.

With the development of PCI technology, the 7F guided catheter and a 7F radial artery sheath have been increasingly widely used. Using a standard 7F sheath *via* the radial artery will cause significant sheath-radial artery mismatch in a considerable number of patients, resulting in a low puncture success rate, pain, RAO, and other complications. To solve the abovementioned problems, we ensured that the thin-walled sheath emerged. Aminian et al. reported their experience using a 7F radial artery thin-walled sheath in complex trans-radial PCI, showing a higher procedural success rate and a lower incidence of vascular complications (8). There are few studies on PCI therapy with a 7F thin-walled sheath placed *via* dTRA. This study intends to demonstrate the safety and effectiveness of PCI with a 7F thin-walled sheath placed *via* dTRA and its ability to provide safe and feasible vascular access for complex coronary intervention.

## Materials and methods

### Study design

This was a single-center retrospective observational study. A total of 102 patients who received complex PCIs with a 7F thin-walled sheath placed *via* dTRA in the catheter room of our hospital from May 2020 to October 2021 were selected as the subjects. According to the operator’s expertise, patients with complex coronary interventional procedures [such as left main coronary artery disease, bifurcation lesion, jailed balloon technique, percutaneous transluminal coronary rotational atherectomy (PTCRA), and chronic occlusive lesion] for whom a 7F guided catheter was required were eligible. All patients were treated with 7F radial artery thin-walled sheaths (APT Medical, China) and were followed up 30 d after the procedure by ultrasound examination of the radial artery.

The study was performed by an experienced operator (who had performed more than 200 dTRA procedures), with the ultrasound examination and interpretation performed by two experienced physicians. Each subject signed an informed consent form, and the ethics committee approved the study of the hospital.

### Exclusion

The exclusion criteria were as follows:

(1) patients with an ulnar artery occlusion possible after an Allen test (+),
(2) patients without a palpable pulse of the distal radial artery,
(3) patients suffering from being hemodynamically unstable,
(4) patients who refused to sign the informed consent form, and
(5) patients who failed to complete the 30-d follow-up.

### Study procedure

#### Color doppler ultrasound imaging

**FIGURE 1:**
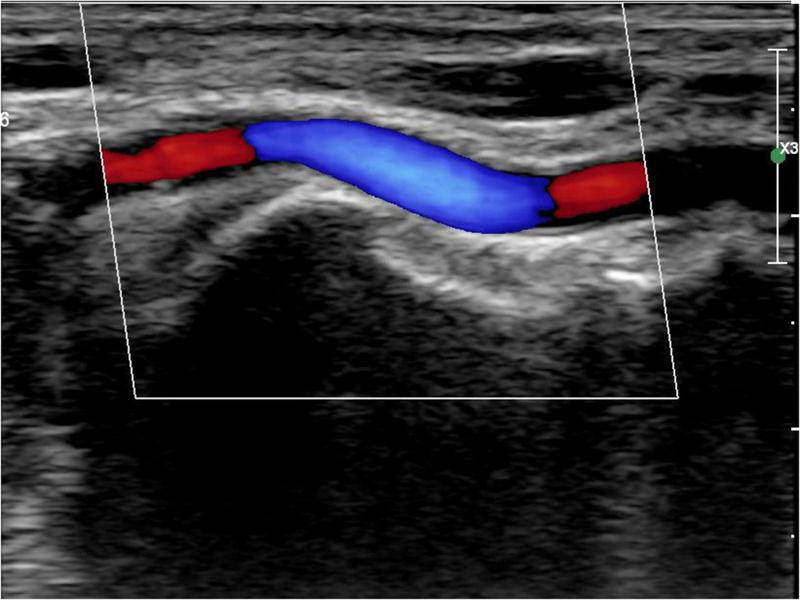
Distal radial artery ultrasound.

All patients were followed up 1 d before, 1 d after, and 30 d after the procedure for radial artery ultrasound examination (9). A Philips Epiq 7c (Koninklijke Philips N.V., the Netherlands) was used for color Doppler ultrasound with linear probe L12-3. The distal radial artery diameter and cross-sectional area were measured (Figure 1). The definition of RAO was that there were no obvious blood flow signals in the radial artery lumen on a color Doppler ultrasound examination. A distal RAO was defined as a flow reversal on a color Doppler ultrasound in the radial artery in an anatomical snuffbox. All data were measured three times and averaged. Two experienced physicians performed color Doppler ultrasound examinations and interpretations of all patients.

#### Sheath description

APT Medical’s 7F Braidin^®^ sheath is thin-walled. It can accommodate any 7F-guided catheter with an inner diameter of 2.46 mm and an outer diameter of 2.72 mm. The reason for its small outer diameter is that the wall thickness is only 0.005 inches, one-half of the wall thickness of the commercially available models (10). The inner and outer diameters of commonly used 6F and 7F radial sheaths are shown in Table 1.

**TABLE 1:** Inner and outer diameters of commonly used 6 and 7F radial sheath.

	Inner diameter (mm)	Outer diameter (mm)	Size of guide catheter, which can accommodate (French)
APT Braidin 6F	2.18	2.44	6
Terumo Glidesheath Slender 6F	2.21	2.46	6
Terumo RADIFOCUS 6F	2.21	2.62	6
Corids 6F	2	2.67	6
APT Braidin 7F	2.46	2.72	7
Terumo Glidesheath Slender 7F	2.55	2.79	7
Terumo RADIFOCUS 7F	2.55	2.95	7
Corids 7F	2.33	3.0	7

#### Puncture and hemostasis procedure

**FIGURE 2:**
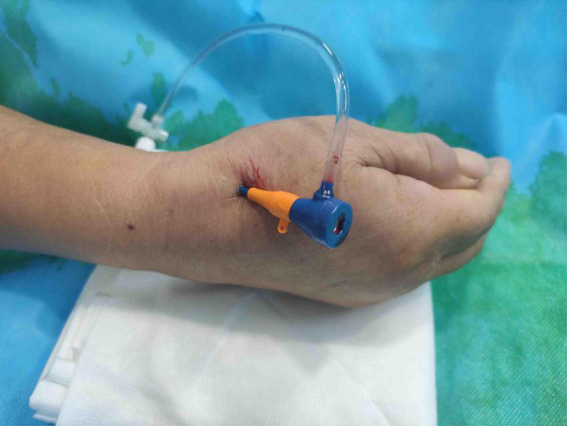
7F thin-walled sheath placed *via* distal transradial artery.

**FIGURE 3:**
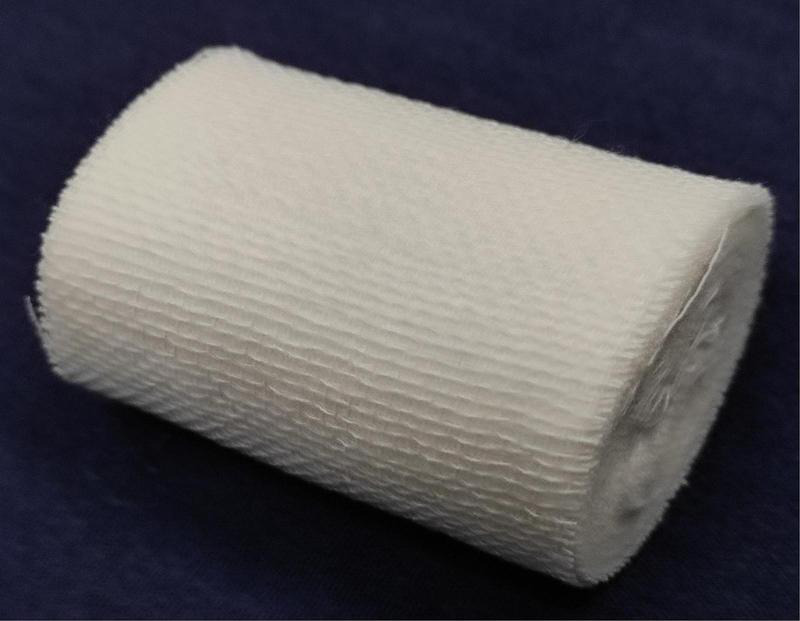
Gauze bandage for hemostasis.

**FIGURE 4:**
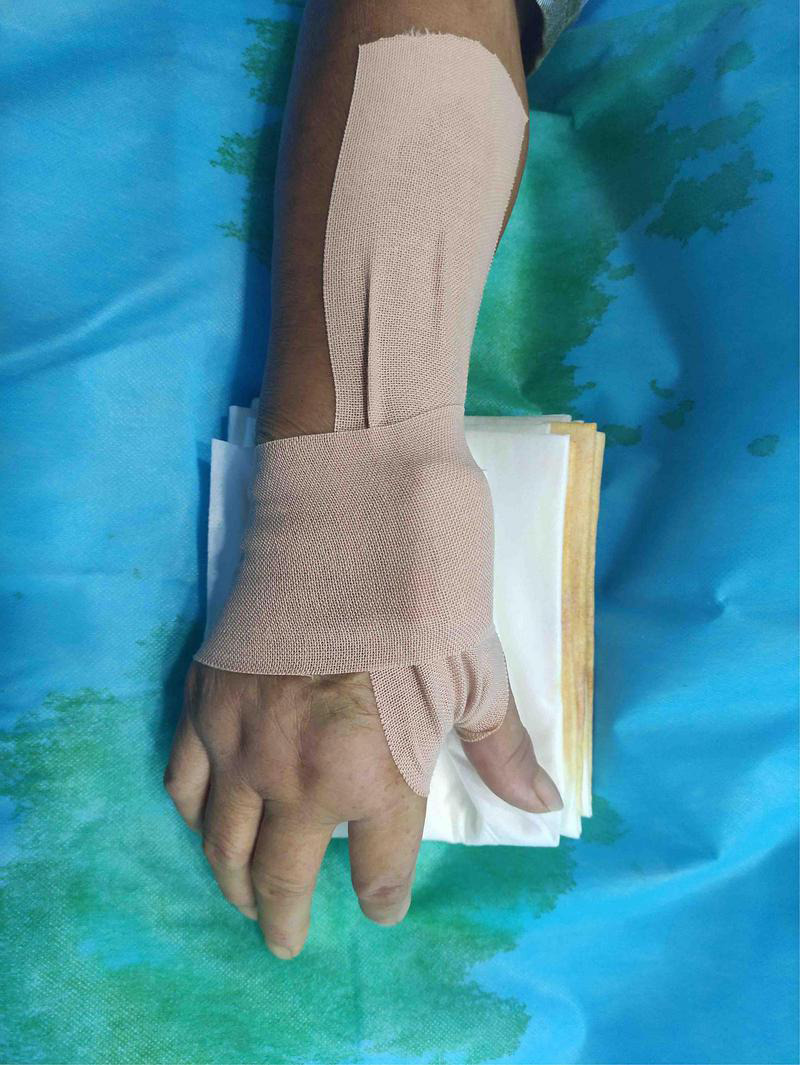
Compression upon distal transradial artery for hemostasis.

**FIGURE 5:**
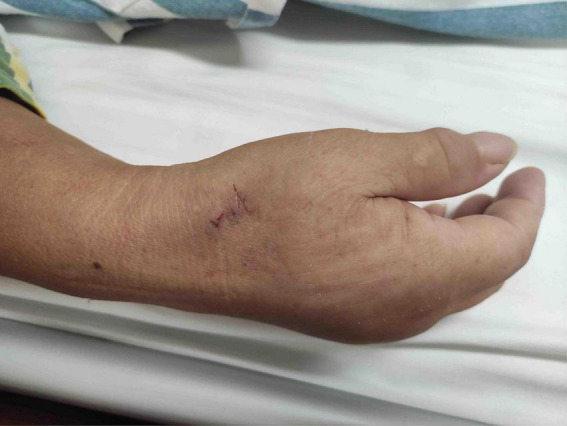
Compression upon distal transradial artery for hemostasis.

The patient’s anatomical snuffbox area was palpated, and the puncture was performed at the site where the distal radial artery pulsated significantly. The “anatomical snuffbox area” refers to a depression in a triangle formed by the tendons of the abductor pollicis longus, extensor pollicis longus, extensor pollicis brevis, and the radial styloid process. There was a palpable pulse in the distal radial artery in the snuffbox area. Local anesthesia with 0.5 mL of Lidocaine was performed using Seldinger’s puncture method. If the operator predicted the presence of coronary artery lesions requiring complex PCI, a 22G puncture trocar (APT Medical, China) was punctured from the lateral to the medial 30°–45° toward the traditional radial artery (wrist). After a successful puncture, the 0.025-inch hydrophilic-coated guidewire was pushed into the artery as a guiding track, followed by the placement of the 7F Braidin^®^ sheath (APT Medical, China) along the guidewire (Figure 2). If the operator did not predict any coronary artery lesions, a 20G puncture trocar (Terumo Corp., Tokyo, Japan) was first used for the puncture as described above. After a successful puncture, a 0.021-inch hydrophilic-coated guidewire was pushed into the artery as a guiding track, followed by the placement of a 6F Radifocus^®^ II sheath (Terumo Corp., Tokyo, Japan) along the guidewire and coronary angiography. A 7F Braidin^®^ sheath (APT Medical, China) was replaced with a 0.025-inch hydrophilic-coated guidewire after the operator determined that intervention therapy with a 7F guided catheter was required for the coronary lesion. The routine injection of 500 μg of nitrene and heparin along the arterial sheath was performed; 5,000 IU of heparin was used for CAG, and heparin (120 IU/kg) was added into the arterial sheath for PCI, according to the patient’s body weight, to maintain the activated clotting time at 250–300 s. The radial artery sheath was removed after the procedure, and a gauze bandage (Jianqi Medical Equipment Corp., China), which was made of rolled-up gauze (Figure 3), and two elastic tapes were used as a cross-compression dressing (Figures 4, 5). Puncture failure was defined as the failure to puncture the distal radial artery after five attempts.

Decompression was performed 4 h after the procedure to check whether the bleeding continued. If there was no bleeding, the radial compression device (RCD) could be completely removed. If the bleeding continued, the gauze was pressed and fixed again; it was also checked every 10 min; the RCD was removed after the bleeding stopped.

The puncture success rate (the percentage of the successful insertion of the sheath into the radial artery), the number of cases of direct placement and 7F/6F sheath exchange placement, the number of cases of the left and right distal radial artery, the time of artery punctures (the time from the first local infiltration anesthesia to the sheath placement), and the time until RCD removal (the time from the sheath removal immediately after the procedure to the complete removal of the RCD) were recorded.

### Procedural characteristics

The success or failure of procedures in patients with a successful puncture was recorded. Emergency/elective PCI status, complex PCI (such as left main coronary artery diseases, bifurcation lesions, the jailed balloon technique, PTCRA, and chronic occlusive lesions), operation time, exposure time, exposure dose, the dosage of contrast agent, and the use of Bivalirudin were also recorded.

### Pain score (numeric rating scale) and complications

Different pain intensities were marked on a scale of 0–10, and the patient identified the index when the pain was most severe. The scale ranged from 0 for no pain to 10 for the most severe pain. A score below 4 indicated mild pain (not affecting sleep), 4–7 indicated moderate pain, and above 7 indicated severe pain (inability to sleep or waking up from sleep) (11).

Complications included the following: (1) local hematomas [graded according to the EASY criteria: type I is ≤ 5 cm diameter, type II is ≤ 10 cm diameter, type III is > 10 cm but not above the elbow, type IV extends above the elbow, and type V is anywhere with ischemia of the hand (12)]; (2) RAO; (3) arteriovenous fistula; (4) pseudoaneurysm; (5) radial artery perforation; (6) radial artery dissection; (7) radial artery spasm (spasm is defined as pain perceived by the patient and/or difficulty perceived by the operator during insertion, manipulation, and/or withdrawal of the introducer sheath or catheter); (8) sheath kinking (major sheath kinking was defined as a significant deformation of the sheath leading to vascular complication and/or procedural failure); and (9) nervous injury (abnormal sensation and activity of the dorsoradial half, radial two-half fingers, upper arm, and the back of the forearm, or even wrist drop in severe cases).

### Statistical analysis

Statistical analysis was performed using IBM^®^ SPSS™ Statistics v22.0 software. Measurement data were expressed as mean ± standard deviation. Enumeration data were expressed as a percentage. Student’s *t*-test and the Kruskal–Wallis test were used to compare measurement data, and a Chi-squared test or Fisher’s exact test was used to compare enumeration data. A value of *P* < 0.05 indicated that the difference was statistically significant.

## Results

### Baseline characteristics

A total of 102 patients who received complex PCIs with a 7F thin-walled sheath placed *via* dTRA in the catheter room of the subject hospital from May 2020 to October 2021 were selected as the subjects after exclusion according to the exclusion criteria. The demographics are shown in Table 2.

**TABLE 2:** Baseline characteristics of all patients.

Baseline characteristics	*n* = 102
Age, y	63.2 ± 10.1
Female/male	31/71 (30.4/69.6)
Weight (kg)	69.8 ± 9.6
Height (cm)	166.9 ± 7.2
BMI cardiovascular risk factors	25.0 ± 2.7
Hypertension, *n* (%)	64 (62.7)
Diabetes mellitus, *n* (%)	29 (28.4)
Dyslipidemia, *n* (%)	22 (21.6)
Smoking history	36 (35.3)
Prior myocardial infarction, *n* (%)	17 (16.7)
Prior stroke, *n* (%)	12 (11.8)
PCI history CABG history Acute coronary syndrome	45 (44.1) 10 (9.8) 31 (15.3)
Heart failure with EF < 40%	11 (10.8)
Creatinine clearance (mL/min)	87.5 ± 20.1
Medications (30 days after PCI)	
Ticagrelor, *n* (%)	77 (75.5)
Clopidogrel, *n* (%)	25 (24.5)
Dual antiplatelet plus anticoagulant therapy, *n* (%)	9 (8.8)
Warfarin, *n* (%)	3 (2.9)
Direct oral anticoagulant, *n* (%)	6 (5.9)

### Operation data

A puncture was performed successfully in 92 out of 102 patients (90.2%). The puncture failed in ten patients: the blood return in the puncture needle was unsatisfactory in six patients; the blood return was good in three patients, but the wire was not inserted successfully; the wire was inserted successfully, and the sheath was not placed successfully in one patient. Among the 92 patients with a successful puncture, a 7F sheath tube was placed directly into 28 patients. A 6F sheath tube was initially placed and then replaced with a 7F sheath after coronary angiography in 64 patients. Right dTRA was used in 82 patients, and left dTRA was used in 10 patients. The procedures were performed successfully in 90 patients (97.8%) and were unsuccessful in two patients, both of whom suffered from chronic occlusive lesions. There were 17 emergency PCIs and 75 elective PCIs. There were 31 cases of left main coronary artery disease, 59 cases of bifurcation lesions, 53 cases of jailed balloon technique performed, 10 cases of PTCRA applied (three cases with a maximum 1.75 mm rotary grinding head and one case with a maximum 2 mm rotary grinding head), and 23 cases of chronic occlusive diseases. Bivalirudin anticoagulation was used in 21 cases. The average operation time and exposure time were 122.3 and 37.3 min, respectively. The average exposure dose was 3,150.0 mGy, the average dose of contrast agent was 167.7 mL, and the average puncture time and RCD removal time were 4.2 and 254.8 min, respectively (Table 3).

**TABLE 3:** Procedural characteristics.

	*n* = 92
Direct placement of 7F sheath	28 (30.4%)
Right dTRA *n* (%)	82 (89.1%)
Successful operation *n* (%)	90 (97.8%)
Emergency operation *n* (%)	17 (18.5%)
Left main coronary artery disease *n* (%)	31 (33.7%)
Bifurcation lesions *n* (%)	59 (64.1%)
Jailed balloon *n* (%)	53 (57.6%)
Rotational ablation *n* (%)	10 (10.9%)
Chronic occlusive diseases *n* (%)	23 (25.0%)
Application of bivalirudine *n* (%)	21 (22.8%)
Surgery duration (min)	122.3 ± 43.1
Exposure duration (min)	37.3 ± 15.0
Exposure dose (mGy)	3150.0 ± 1238.7
Contrast agent dosage (mL)	167.6 ± 48.1
Puncture time (min)	4.2 ± 1.9
Time of removal of RCD (min)	254.8 ± 36.6

### Ultrasonic examination results

A color ultrasound examination was performed on 92 patients who received successful distal radial artery puncture 1–30 d after the procedure. This revealed two cases of RAO, both located in the forearm segment of the radial artery, and three cases of distal RAO, all located in the puncture site of the distal radial artery. There were no statistically significant differences in the diameter and area of the radial artery lumen and the diameter and area of the distal radial artery lumen at 1 d before, 1 d after, and 1 month after the procedure (Table 4).

**TABLE 4:** Vascular ultrasound results before and after dTRA intervention.

	Preoperative	1-day after procedure	1-month after procedure
RAO, *n* (%)	0	2 (2.2%)	2 (2.2%)
DRAO, *n* (%)	0	3 (3.3%)	3 (3.3%)
Diameter of the radial artery lumen	2.62 ± 0.49	2.76 ± 0.62	2.56 ± 0.67
Area of the radial artery lumen	6.49 ± 2.57	8.15 ± 10.18	6.72 ± 3.58
Diameter of the distal radial artery lumen	2.17 ± 0.44	2.16 ± 0.55	2.06 ± 0.56
Area of the distal radial artery lumen	4.00 ± 1.98	4.17 ± 2.08	3.88 ± 2.22

RAO, radial artery occlusion; DRAO, distal radial artery occlusion. Compared with that before the procedure, *^a^p* < 0.05. Compared with that 1 day before the procedure, *^b^p* < 0.05.

### Pain score and complications

The mean and maximum pain scores of 92 patients after a successful distal radial artery puncture were 2.2 and 6, respectively. There were five cases (5.4%) of grade 1–2 local hematoma. Radial artery spasm and nervous injury occurred in two patients (2.2%), and arteriovenous fistula occurred in one patient (1.1%). Radial artery perforation, radial artery dissection, pseudoaneurysm, and sheath kinking did not occur (Table 5).

**TABLE 5:** Pain score and complications.

	*n* = 92
NRS score	2.2 ± 1.1
Local hematoma *n* (%)	5 (5.4%)
Perforation of radial artery *n* (%)	0 (0%)
Dissection of radial artery *n* (%)	0 (0%)
Pseudoaneurysm *n* (%)	0 (0%)
Arterio-venous fistula *n* (%)	1 (1.1%)
Radial artery spasm *n* (%)	2 (2.2%)
Sheath kinking *n* (%)	0 (0%)
Nervous injury *n* (%)	2 (2.2%)
Radial artery occlusion *n* (%)	2 (2.2%)
Occlusion of the distal radial artery *n* (%)	3 (3.3%)

## Discussion

This single-center retrospective study aimed to investigate the efficacy and safety of PCI using a 7F thin-walled sheath placed *via* dTRA. The results showed that dTRA had a higher puncture success rate and procedural success rate, which was safe and effective for PCI. In addition, it was characterized by short postoperative hemostasis time, relatively mild pain, fewer puncture-related complications, and a low RAO rate.

In traditional TRA diagnosis and treatment, RAO of the forearm is a common and important complication. The incidence of RAO was 1–10% in patients who received transcranial arteriography and/or PCI and was even higher in patients who received repeated TRA or emergency intervention, making re-intervention difficult (13). In 2011, Babunashvili and Dundua (14) reported RAO at the proximal reverse opening of dTRA *via* the anatomical snuffbox in two patients, introducing dTRA into the field of intervention.

The clinical study of Kiemeneij (4) in 2017 confirmed the safety and feasibility of dTRA intervention. The puncture point of dTRA is located at the wrist, which causes minor damage to the radial artery of the forearm and reduces the occurrence of forearm RAO. All clinical studies on dTRA concluded that the low incidence of forearm RAO was a major advantage of this access. Kiemeneij (4) performed left dTRA angiography in 70 patients (25 of them received PCI *via* the same access), and ultrasound examination before discharge showed that RAO did not occur in the forearm of any patient. Further, only one patient suffered distal RAO in the anatomical snuffbox.

Guering et al. (15) reported that, compared with conventional radial artery access, dTRA could prevent proximal RAO 24 h and 30 d after the procedure. According to Mizuguchi et al. (16) an ultrasound examination performed 1 month after interventional treatment *via* dTRA showed that forearm RAO only occurred in 1 out of 228 patients (0.4%). In this study, 7F radial artery sheaths were applied to all patients, and the incidence of radial and distal RAO was relatively low after 1 day and 1 month after the procedure.

The possible reasons include the following: (1) the puncture site for dTRA was located between the proximal branch supplying the superficial palmar arch and the distal short periosteum artery, so even after the puncture site was occluded, the downstream flow through the superficial palmar arch could be maintained, which could reduce the risk of retrograde thrombosis in the radial artery of the forearm (17); (2) the 7F Braidin^®^ sheath of APT Medical had an outer diameter of 2.72 mm, which is only 0.1 mm larger than Terumo’s 6F Radifocus II sheath, and it had a hydrophilic coating so that it could minimize the occurrence of vascular complications, such as RAO; and (3) the compression site was located at the distal radial artery more superficially, so gauze and elastic tape could fully stop the bleeding without a special RCD. The low pressure required for hemostasis and the short compression time made it difficult to form a vascular occlusion.

In addition to RAO, complications at puncture sites, such as pain and hematoma, were also common problems that required appropriate attention. Improper management of hematoma compression in patients receiving traditional TRA could lead to hematoma, nerve palsy, hand dysfunction, amputation, and other adverse consequences. However, the puncture site of dTRA is located at the wrist, where the radial artery is shallow and small in diameter. Additionally, there is bony structural support in the deep puncture site, so it is convenient to stop bleeding by compression, and it is conducive to reducing the occurrence of hematoma and other complications at the puncture sites.

Antonios et al. (18) observed 44 patients who received interventional treatment *via* dTRA and found that hematoma of EASY grade III or higher did not occur in any patient after the procedure. This study’s average pain score and complication incidence were lower. A possible reason may be that it was essential to bypass the thumb in the use of the local cross-dressing method with gauze and elastic tape. Hence, the position was relatively stable, and displacement was not likely to occur, reducing the risk of bleeding and hematoma. Because of the small compression range and the small range of influence on the venous return, a secondary pressure rise after compression seldom occurs, so it will rarely cause blisters and ecchymosis, thus effectively reducing the pain of patients. It is worth mentioning that sheath kinking did not occur in any patients in the present study, which was due to the strong support of the three-layer sheath structure reinforced by the steel mesh of the Braidin^®^ vascular sheath.

Through the development of PCI technology, an increasing number of complex lesions can be treated with PCI. Although currently used for most PCIs, a 6F guided catheter has limited access and has even led to the failure of PCI for some complex pathological changes, such as the lesions for which a cutting, jailed, or kissing balloon is required, the lesions for which a large-diameter rotational ablation head is required, and chronic occlusive lesions.

Therefore, it is necessary to use a 7F guiding catheter and a 7F radial artery sheath in a considerable number of PCI procedures. Moreover, about 29–67% of men and 60–85% of women have a radial artery diameter less than the outer diameter of a standard 7F radial sheath (19–21). Thus, the placement of a standard 7F sheath *via* a radial artery will result in a significant sheath–radial artery mismatch in a significant number of patients, leading to local inflammation, reduced blood flow, spasms, chronic remodeling, and, ultimately, RAO. To overcome these anatomical limitations, we ensure that the thin-walled sheath for improved radial artery access has been developed. Its inner diameter is compatible with the 7F guided catheter, and its outer diameter is smaller than the standard 7F sheath.

Aminian et al. reported their experience using a 7F radial artery thin-walled sheath manufactured by Terumo Corporation, Japan, in complex trans-radial PCI. They found that such a sheath could lead to a higher procedural success rate and a lower incidence of vascular complications (8). APT Medical’s 7F Braidin^®^ sheath, which was used in this study, is a thin-walled sheath with a hydrophilic coating. The inner diameter allows it to accommodate any 7F guided catheter, and its outer diameter is 2.72 mm. The reason for its small outer diameter is that its wall thickness is only 0.005 inches, only one-half of that of the commercially available models. The puncture success rate was 90.2% among the 102 patients in this study, with a high success rate. Nigopan et al. reported that ultrasound-guided arterial catheterization was superior to the standard palpation technique (22). However, because the operator had not received training in ultrasound-guided puncture and the capacity of ultrasound doctors was limited, the ultrasound-guided puncture was not routinely used in this study.

The 6F sheath was replaced with a 7F sheath with the help of a guidewire in some patients. Replacement of a 6F sheath with a 7F sheath was available to all patients. This was closely related to the above characteristics of the 7F Braidin^®^ sheath. Previous studies have shown that distal radial artery access is safe for PCI in patients with acute coronary syndrome and complex lesions, in addition to meeting the requirements for routine coronary intervention. This may include complex multivessel PCI (23), unprotected left main artery PCI supported by intravascular ultrasound (24), and multivessel PCI for elderly patients with acute coronary syndrome (25).

There were 17 emergency PCIs and 75 elective PCIs in the present study. There were 31 cases of left main coronary artery disease, 59 cases of bifurcation lesions, 53 cases where the jailed balloon technique was performed, and 10 cases of PTCRA. The procedure success rate was 97.8%, covering the most common and complex PCI procedures. The high success rate, the short average puncture time, and the short RCD removal time demonstrate the feasibility of PCI with the placement of a 7F thin-walled sheath *via* dTRA.

It is particularly important to note that 10 patients who underwent PCI *via* a left distal radial artery were also included in this study, as dTRA could provide the operator with a better ergonomic position in the interventional therapy *via* the left hand. The patient’s left hand was placed on the abdomen, and the operator could operate on the right side of the patient as usual, which could reduce not only the operator’s exposure to radiation but also the operator’s fatigue and the patient (26).

Gabriele et al. reported their experience with left dTRA for coronary chronic total occlusion interventions using a 7F Glidesheath Slender^®^, demonstrating that left dTRA in the setting of chronic total occlusion PCI using this sheath is feasible and safe, with a high procedural success rate and low access-site complication rates (27). Wang et al. reported that the distal radial artery approach had a lower rate of RAO, indicating that it could be used as an alternative to the classic radial artery approach (28). Hammami et al. reported that the distal radial approach was feasible and safe for coronary angiography and interventions (29). Comparisons between the end-points of the present study and the above studies are shown in Table 6.

**TABLE 6:** Comparison between end-points of the present study and other studies.

Study by	Journal/year	Research question				Main outcomes	
			Puncture success rate (%)	Radial artery spasm rate (%)	Distal radial artery occlusion rate (%)	Radial artery occlusion rate (%)	Sheath kinking rate (%)
The present study	/	Safety and feasibility of 7F thin-walled sheath *via* distal transradial artery	90.2	2.2	3.3	–	0
Gabriele L. Gasparini’s study	EuroIntervention /2019	Safety and feasibility of 7F Glidesheath Slender *via* left distal transradial artery	82.9	0	4.3	–	0
H. Wang’s study	Ann. Palliat. Med./2020	Comparison of the clinical effects and safety between the distal radial artery and the classic radial artery approaches	94.9	2.9	1.9	5.2	–
R. Hammami’s study	Libyan J. Med. /2021	Comparison of feasibility and safety between distal radial approach and conventional radial approach	95.1	6	0	3.1	–

This study was not without its limitations: (1) this was a single-center retrospective observational study with small sample size; (2) there was no conventional radial artery puncture group for control analysis; (3) ultrasound-guided puncture was not routinely used in this study; and (4) the follow-up time was short. Compared with conventional radial artery puncture, the advantages and disadvantages of the approach here should be verified by further large-scale randomized controlled clinical studies.

## Conclusion

In conclusion, PCI with the placement of a 7F thin-walled sheath *via* dTRA is characterized by a high puncture success rate, a high procedure success rate, a low postoperative RAO rate, and a low incidence of local hematoma and other complications, making it safe and feasible.

## Data availability statement

The original contributions presented in this study are included in the article/supplementary material, further inquiries can be directed to the corresponding author/s.

## Ethics statement

The studies involving human participants were reviewed and approved by the Xuzhou Central Hospital. The patients/participants provided their written informed consent to participate in this study.

## Author contributions

BZ, C-GF, and BH: conception and design of the research. BZ and YL: acquisition of data and statistical analysis. BZ and BH: analysis and interpretation of the data. C-GF: obtaining financing. BZ and C-GF: writing of the manuscript. YL and C-GF: critical revision of the manuscript for intellectual content. All authors read and approved the final draft.
